# Bioactive compounds enrichment in rabbit doe’s diet pre-and during pregnancy improves productive and reproductive performance and cost-effectiveness under hot climates

**DOI:** 10.1007/s11250-024-04166-w

**Published:** 2024-10-12

**Authors:** Mohamed E. El-Speiy, Moustafa M. Zeitoun, Mohamed A. El-Sawy, Tarek A. Sadaka, Bahaa M. Abou-Shehema, Mohamed M. Abdella, Hossam A. Shahba, Mahmoud R. Habib

**Affiliations:** 1 Animal Production Research Institute, Agriculture Research Center, Giza, Egypt; 2 Department of Animal and Fish Production, Faculty of Agriculture, Alexandria University, Alexandria, Egypt

**Keywords:** Rabbit does, Synbiotic, Bee pollen, Date palm pollen, Production, Reproduction, Heat stress

## Abstract

This study aimed to investigate the effect of diet supplementation with a symbiotic (SY), bee pollen (BP), honey bee (HB), date palm pollen (DPP) and their mixture (MIX) on female rabbit productive and reproductive performances under desert hot climates. Seventy-two Californian does of 5 months age and average body weight of 3250 ± 78.2 g were randomly allotted into six groups, each of 12 does. All does orally receive 3 ml distilled water for 10 days before mating and 28 days during pregnancy. Treatments were repeated for four consecutive parities. The first group served as control (C) given distilled water only, however the second, third, fourth and fifth groups were supplemented with 3 ml distilled water containing 0.2 ml SY, 200 mg DPP, 200 mg BP, 0.2 ml HB/doe per day, respectively. While, the sixth group does were given all previous ingredients (MIX). Sexual receptivity rate, fertility rate, kindling rate, and newborn traits were recorded. Also, maternal feed intake, feed conversion ratio, and digestibility coefficients of nutrients were recorded. Does in all groups were artificially inseminated with 0.5 ml of fresh heterospermic semen of 15 fertile bucks extended in Tris at 806–1006 sperm/ml. Treatment increased maternal body weight and daily gain with highest values (*P* < 0.05) in BP, SY, and MIX does. All treatments enhanced feed intake and feed conversion ratio (FCR) compared with control. Number services per conception decreased (*P* < 0.01), while litter size and weight and survival at birth and weaning increased (*P* < 0.01) in treated than control does. Treated does produced more milk than control. Digestibility coefficients of all nutrients were improved (*P* < 0.01) in treated does. In conclusion, supporting rabbit does pre- and during pregnancy with diets supplemented with a mixture of honey bee, date palm pollen, bee pollen, and synbiotic improves the productive and reproductive performances of rabbit does and their offspring.

## Introduction

Due to the threats of pandemics and wars in the world, there emerged an acute shortage of grains and feedstuffs for human and livestock feeding. Therefore, researchers struggled to find out a new trend of additives to compensate for the lack of nutrients and to maintain healthy and productive livestock. The nutritional composition of medicinal plants is believed to benefit sexual performance and fertility, and these plants can be successfully used to treat sexual dysfunction or to serve as fertility-enhancing agents (Sumalatha et al. 2010). Moreover, consumer awareness of food safety is expanding rapidly; for instance, health concerns increased over the use of antibiotics as growth promoters in farm animal feed (De Marco et al. 2015). On the other hand, interest in natural alternative animal feeding strategies has remained fairly steady among researchers (Hosseini et al. 2013). It has been recently focused on some bioactive ingredients to improve the productivity and reproductive performances of different animal species. These bioactive ingredients include; bee pollen (BP) (Abdelnour et al. 2018, Nowosad et al. 2022), honey bee (Lika et al. 2021), date palm pollen (Saleh et al. 2021), probiotics (Melara et al. 2022), and synbiotic (Gibson and Roberfroid 1995). The significant bioactive compounds in bee pollen (BP)are; flavonoids, polyphenols, carbohydrates, enzymes, vitamins, fatty acids, essential amino acids and carotenoids which depend on the botanical and geographical origin (Denisow and Denisow-Pietrzyk 2016). Date palm pollen (DPP) is known to include a variety of compounds; including high phenolic content, flavonoids, and anthocyanins, and a considerable amount of selenoproteins (Baliga et al. 2011), making it an excellent candidate for antioxidant activity with little negative side effects (Fallahi et al. 2015). Honey bee (HB) contains a high amount of metabolizable energy in the form of glucose and fructose, and also antibacterial activity against microorganisms that inhibits a broad spectrum of bacteria, including aerobes and anaerobes, Gram positives and negatives (Hannan et al. 2009). It is well known that heat stress imposes negative impacts on the productive, reproductive, antioxidant, and immunity potential of rabbits (Liang et al. 2022), goats (Sejian et al. 2021), sheep (McManus et al. 2020), and cattle (Herbut et al. 2019). Therefore, this study aimed to investigate the ameliorative effects of some bioactive compounds given before and during pregnancy on the productive and reproductive performance and economic feasibility of rabbit does that raised outdoor under hot condition.

## Materials and methods

The present study is conducted at a private rabbit farm in Qalyuobia Governorate, where temperatures ranged from 29 to 35 °C, relative humidity of 43 to 65%, and light/dark cycles of 16: 8 h throughout the day.

## Basal diet

Table 1 presents the ingredients and the chemical analysis of the basal diet.

**Table 1:** Composition and chemical analysis of basal diet

Ingredients	%	Calculated analysis			
Yellow corn	6.22	Crude protein, %			18.8
Soybean meal, 44%	22.33	Crude fiber, %			13.0
Wheat bran	23.33	Ether extract, %			3.0
Barley	15.00	Digestible energy (Kcal/kg diet)			2680
Alfalfa hay	30.12	n-6 poly unsaturated FAs%			0.3
Ground limestone	1.00	n-3 poly unsaturated FAs%			1.03
		Zn, ingredients + Premix (50 + 50 mg)			100
Dicalcium Phosphate	1.20	***Determined analysis (g/kg diet)***			
Sodium chloride	0.50	Dry matter	897.1	Crude fiber	138.5
Vit. + min. premix^*^	0.30	Organic matter	801.4	Ether extract	26.2
Total	100	Crude protein	169.8	Nitrogen-free extract	575.0
				Ash	87.9

*Each 3 kg of premix contains: Vit. A: 12,000,000 IU; Vit.D_3_: 3,000,000 IU; Vit. E: 10.0 mg;

Vit. K_3_: 3.0 mg; Vit. B_1_: 200 mg: Vit. B_2_: 5.0 mg; Vit.B_6_: 3.0 mg: Vit. B_12_: 15.0 mg; Biotin: 50.0

mg; Folic acid:1.0 mg; Nicotinic acid: 35.0 mg: Pantothenic acid: 10.0 mg; Mn: 80 g; Cu: 8.8 g;

Zn: 50 g; Fe:35 g; I: 1 g; Co: 0.15 g and Se: 0.3 g

## Experimental design

Seventy-two Californian does with age of around 5 months and average body weight of 3250 ± 78.2 g were used in the experiment. Rabbit females were randomly allotted into 6 groups (*n* = 12 does/group) and orally received 3 ml distilled water as a base solution containing different tested compounds for 10 days before mating and 28 days after mating during pregnancy, and the experiment was repeated for 4 consecutive parities.

The first group served as a control (**C**) given 3 ml distilled water per each doe. The second, third, fourth, and fifth groups were supplemented with 0.2 ml synbiotic (**SY**), 200 mg date palm pollen **(DPP)**, 200 mg bee pollen (**BP**), and 0.2 ml honey bee **(HB)** per doe/day during treatment period, respectively. While, the sixth group does were given 0.2 ml SY + 200 mg DPP + 200 mg BP + 0.2 ml HB per doe/day (**MIX**)*.* The does were artificially inseminated for the first time at age of 5 months using a pool of semen collected from 15 fertile bucks. The inseminated does were tested for pregnancy by abdominal palpation. Ten days after kindling the does were reinseminated. The doses of BP and DPP used in this trial were according to El-Hanoun et al. (2007), while the synbiotic (SY) dose followed Sherief et al. (2012). Synbiotic was commercially purchased from Bactizad company, Egypt and has a trade name called ZAD (anaerobic bacteria).

## Housing and management

Rabbit does were housed outdoor in galvanized wire batteries, approximately 60 × 55 × 40 cm (L × W × D). The batteries were equipped with pellet feeders and automatic drinkers. Fresh water was provided for animals used in the experiment. The basal experimental diet was designed and pelleted to satisfy the nutritional requirements of rabbits (crude protein 18.8%, crude fibers 13.0%, ether extract 3.0% and digestible energy 2680 kcal/kg diet) according to NRC 1977.

## Artificial insemination of does

Does were inseminated using a fresh diluted heterospermic pooled semen (1 Semen: 3 extender) collected from 15 fertile bucks. Artificial insemination (AI) was achieved by depositing 0.5 ml of freshly diluted semen deeply in the region of the vagina by a sterile catheter. Semen ejaculates were individually evaluated microscopically, and the ejaculates that exhibited active progressive motility percentages of > 70% were pooled and extended in TRIS extender. The final concentration of motile sperm/ml was 80–100^6^. The insemination was immediately followed by administration of 0.8 µg (IM) of Buserelin (0.2 ml Receptal; GnRH analog; Hoechst, Frankfurt, Germany) to stimulate ovulation (Lopez and Alvariño, 2000).

## Reproductive traits

### Sexual receptivity rate

Number of dorsiflexion's of a vasectomized buck in response to mounting was recorded to calculate receptivity. According to the International Rabbit Reproduction Group (IRRG) (2005), does were evaluated in the presence of a vasectomized buck by dividing the numbers of dorsiflexion on the total number of mounts × 100 (Avitsur and Yirmiya 1999). All receptive does were artificially inseminated.

## Fertility rate

Fertility was tested on the 14th day post-insemination through abdominal palpation. The fertility rate was calculated as the ratio of the number of conceived does divided on the total number of inseminated does multiplied by 100.

## Kindling rate

Kindling rate was calculated by dividing the number of does that kindled on the total number of inseminated does multiplied by 100.

## Litter size and litter weight at birth

The litter number and weight at birth were determined by counting and weighing pups immediately after kindling. The number of stillborn was taken into account (Paci et al. 2012). The survival rate was defined by dividing the number of pups born and stayed alive at 28 days of age on the total number of pups born.

## Blood collection and plasma harvesting

At the end of the experiment, blood samples (5 ml) were withdrawn from the marginal ear veins in the morning before receiving feed and water. Blood was collected from five does within each treatment using sterile tubes containing heparin. Blood samples were centrifuged at 3000 rpm for 15 min, plasma samples were harvested and kept frozen at -20˚C until used for further analyses.

## Plasma digestive enzymes and thyroid hormones

Amylase and lipase activities were measured in plasma on a Chem-Well 2900 (T) automated analyzer (United States) with the use of the appropriate HUMAN reagent kits (Germany). Amylase activity was determined according to the method mentioned by Gubern et al. (1995). Serum lipase activity was determined by the method of Tietz et al. (1972).

Plasma levels of triiodothyronine (**T**_**3**_) and thyroxin (**T**_**4**_) were analyzed using commercial simple solid phase enzyme-linked immune absorbent assay (ELISA) kits (Diagnostics Test Canada, Inc., Ontario, Canada) according to Odell et al. (1968).

## Digestibility coefficients

Digestion trial was done according to Tassone et al. (2021). Digestibility coefficient was calculated as follows:

$$Digestibility\;coefficient=\left[\left(Intake-excreted\right)/intake\right]\ast100$$

## Milk yield

Milk production from each doe was estimated by weighing the offspring before and after suckling.

### Statistical analysis

Data were subjected to analysis of variance using the SAS program (SAS 2002). The significant mean differences among groups were separated by Duncan's multiple rang test (Steel and Torrie 1980).

## Results

### Productive performance of does and offspring during lactation

Table 2 illustrates the maternal body weight and daily gain after 10 days of mating. All supplemented does expressed significant higher (*P* < 0.01) body weights than the control does. Daily body weight gain during 10 days post breeding was heaviest (*P* < 0.01) in the BP, SY, and MIX does. Additionally, other groups showed higher daily weight gain than control does (*P* ≤ 0.01). Conversely, control does consume more feed (*P* ≤ 0.01) than treated animals. Thus, the feed conversion ratio (FCR) in treated does surpassed its counterpart in control ones. Overall, providing rabbit does with the tested compounds reduced the amount of feed required to gain 1 kg body gain to nearly half that found in control does.

**Table 2:** Effect of bioactive compounds supplementation during pregnancy on body weight, daily gain, and feed intake of Californian rabbit does (*n* = 12/group)

Treatment group	Initial body weight (g)	Body weight 10 d post mating (g)	Weight gain10 d post mating (g)	Daily feed intake (g)	FCR^*^
Control (C)	3168	3400^b^	232^c^	245.4^a^	10.6^a^
Honey bee (HB)	3250	3598^a^	341^b^	234.1^b^	6.87^b^
Date palm pollen (DPP)	3212	3553^a^	348^b^	237.5^b^	6.82^b^
Bee pollen (BP)	3181	3595^a^	406^a^	231.5^c^	5.70^c^
Synbiotic (SY)	3141	3568^a^	427^a^	237.0^b^	5.55^c^
Mixture (MIX)	3189	3619^a^	430^a^	233.5^b^	5.43^c^
SEM	56.87	44.56	4.04	0.8	0.02
*P*-value	NS	0.0001	0.0001	0.0001	0.001

^a^^,^^b,c^Values with different superscripts in the same column differ significantly (*P* ≤ 0.01). NS: Not significant, * FCR: Feed Conversion Ratio = g daily feed intake/g daily gain

Table 3 summarizes data of reproductive traits in control and treated rabbit does. Number of services per conception decreased (*P* < 0.01) due to treatment as compared with control, the reduction approached 30% of the control. Percentage of doe receptivity to the male and percentage of fertilized does increased in treated females by 15–20%. Live litter size and weight at birth, significantly increased (*P* < 0.01) in treated compared with control does. Percentage increase in litter size due to treatment ranged between 28.6–51.7%, with highest live pups’ outcome in does given MIX. Even though, treatment resulted in a significant increase in litter size, mean average pup weight at birth didn’t differ than control.

**Table 3:** Effect of bioactive compounds supplementation on sexual receptivity, number of AIs per conception, fertility percent, alive litter size, and litter weight (n = 12/group)

Treatment group	Receptivity %	No. AIs/conception	Fertility %	Litter size alive at birth (n)	Litter weight at birth (g)
Control (C)	75^b^	1.87^a^	70^c^	5.6^c^	300.0^c^
Honey bee (HB)	90^a^	1.36^bc^	85^ab^	7.4^ab^	410.5^b^
Date palm pollen (DPP)	95^a^	1.42^b^	90^a^	7.2^ab^	422.5^b^
Bee pollen (BP)	95^a^	1.33^bc^	90^a^	8.3^a^	465.0^ab^
Synbiotic (SY)	90^a^	1.40^b^	80^b^	8.4^a^	481.5^a^
Mixture (MIX)	95^a^	1.28^c^	90^a^	8.5^a^	485.5^a^
SEM	3.95	0.09	4.46	0.13	16.56
*P*-value	0.0054	0.0068	0.007	0.0001	0.0089

^a^^,^^b,c^Values with different superscripts in the same column differ significantly (*P* < 0.01)

Table 4 presents data of offspring, maternal milk yield, and milk conversion ratio. Weaning was achieved at age of 28 days. Survival at weaning increased (*P* < 0.01) by about 20% in offspring produced by does that were given the tested compounds than these by control does. Total litter body weight at weaning (@ 28 days of age) of treated does’ offspring exceeded (*P* < 0.01) that derived from control does by 32–72% with the MIX treatment produced the heaviest weight. Moreover, milk yield and milk conversion ratio improved significantly (*P* < 0.01) at weaning in treated does.

**Table 4:** Effect of bioactive compounds supplementation on litter size at weaning, litter survival at weaning, litter weight at weaning, maternal milk yield from day 1 to day 28 per rabbit doe, and milk conversion ratio (*n* = 12/group)

Treatment group	Litter size at weaning (n)	Survival rate %	Litter weight at weaning (g)	Milk yield during 28 days (g)	Milk conversion Ratio (g milk/g gain)
Control (C)	4.1^c^	73.2^b^	2823^d^	3997^b^	1.54^a^
Honey bee (HB)	6.9^b^	93.2^a^	3733^c^	4978^a^	1.48^a^
Date Palm Pollen (DPP)	6.8^b^	94.4^a^	4046^b^	5176^a^	1.42^ab^
Bee pollen (BP)	7.6^a^	92.7^a^	4305^b^	4989^a^	1.28^b^
Synbiotic (SY)	7.8^a^	94.0^a^	4412^ab^	4893^a^	1.23^b^
Mixture (MIX)	8.0^a^	95.2^a^	4880^a^	5256^a^	1.26^b^
SEM	0.146	10.9	30.596	14.98	0 .08
*P*-value	0.0001	0.03	0.0001	0.005	0.002

^a^^,^^b,c^Values with different superscripts in the same column differ significantly (*P* < 0.01)

Data shown in Table 5 refer to the blood plasma levels of thyroid hormones and digestive enzymes in rabbit does. All treatments elevated T3 levels above control. Levels of T3 were higher in DPP, BP, and MIX than HB and SY.

**Table 5:** Effect of bioactive compounds supplementation on T3, T4, amylase, and lipase of rabbit does (*n* = 5/group)

Treatment group	T_3_ (pg/ml)	T4 (pg/ml)	Amylase (U/L)	Lipase (U/L)
Control (C)	35^c^	665^c^	130.66^c^	140.25^c^
Honey bee (HB)	40^b^	1055^a^	149.05^b^	158.40^bc^
Date palm pollen (DPP)	65^a^	830^b^	172.40^b^	184.50^b^
Bee pollen (BP)	65^a^	885^b^	169.00^b^	175.25^b^
Synbiotic (SY)	40^b^	895^b^	164.50^b^	224.30^a^
Mixture (MIX)	65^a^	830^b^	149.95^b^	179.90^b^
SEM	6	57	21.54	14.36
*P*-value	0.006	0.0001	0.0001	0.0001

^a^^,^^b,c,d^Values with different superscripts in the same column differ significantly (*P* ≤ 0.01). *T*_*3*_: Triiodothyronine, *T*_*4*_: Thyroxine

Does given HB exhibited the highest (*P* < 0.01) T4 concentration. Other treatments (DPP, BP, SY. and MIX) revealed similar levels of T4 which were higher (*P* < 0.01) than control.

All treatments increased (*P* < 0.01) the activity of amylase than control by 15–25%.

Does given SY expressed the highest (*P* < 0.01) lipase activity, while does given MIX, BP, DPP, and HB revealed intermediate lipase activities being higher (*P* < 0.01) than control.

Table 6 reveals data of the digestibility coefficients (DC) of nutrients and the nutritive values of feedstuffs supplemented with various bioactive compounds. Digestibility coefficients of dry matter were higher (*P* < 0.01) in treated than control diets by 8–11%.

**Table 6:** Effect of bioactive compounds supplementation on digestibility coefficients of nutrients and nutritive values of rabbit does (*n* = 12/ group)

Treatment group	Digestibility coefficient (%)					Nutritive value		
	*DM	CP	EE	NFE	CF	TDN, %	DCP, %	DE (Kcal/ kg) *
Control (C)	71.92^b^	59.61^c^	58.97^c^	73.61^b^	44.51^c^	61.36^c^	10.25^c^	2718^b^
Honey bee (HB)	78.45^a^	69.87^b^	65.65^b^	78.75^a^	50.43^a^	67.31^a^	11.63^b^	2996^a^
Date palm pollen (DPP)	78.89^a^	71.43^ab^	69.42^a^	79.23^a^	52.21^a^	71.11^a^	13.42^a^	3067^a^
Bee pollen (BP)	79.95^a^	70.63^ab^	67.88^a^	79.84^a^	51.11^a^	68.24^a^	12.15^a^	3023^a^
Synbiotic (SY)	77.30^a^	74.70^a^	68.27^a^	76.42^a^	50.77^a^	73.08^a^	13.01^a^	2894.6^a^
Mixture (MIX)	78.61^a^	73.64^a^	69.88^a^	77.21^a^	51.24^a^	72.12^a^	13.56^a^	3123.1^a^
SEM	0.76	1.22	0.75	0.63	0.75	0.90	0.05	3.75
*P*-value	0.004	0.006	0.003	0.005	0.002	0.007	0.001	0.008

^a^^,^^b,c^Values with different superscripts in the same column differ significantly (*P* ≤ 0.05). *DM*: dry matter, *CP*: crude protein, *EE*: ether extract, *NFE*: nitrogen free-extract, *CF*: crude fiber, *TDN*: total digestible nutrients, *DCP*: digestible crude protein, *DE*: digestible energy

All treatments improved (*P* < 0.01) the DC of crude protein (CP) by 17–24% above control. Moreover, treatments improved DC of ether extract by 12–19% over control. Likewise, starch (NFE) DC improved by 4–8% and DC of crude fiber improved by 14–18% above control.

Nutritive values revealed that TDN was improved by the added compounds by 10–20% with SY resulting in the best value. Similar trend was observed for percentage of crude protein as treatments enhanced their values by 10–30%, keeping that the best treatment was the MIX, followed by DPP. Similarly, digestible energy was highest in case of MIX, followed by DPP and BP and the least improvement was found in HB and SY diets.

Table 7 illustrates data of the economic feasibility of the treatments. The best net revenue was found in SY and MIX. The highest economic efficiency was obtained in SY treatment amounting 6.7 folds more than control.

**Table 7:** Cost effectiveness of the treatment of rabbit does with various bioactive compounds

			Treatment	group			
Item	C	HB	DPP	BP	SY	MIX	Sig
Body weight of doe at weaning, g	3823^e^	3733^f^	4046^d^	4305^c^	4412^b^	4880^a^	**
Litter size at weaning, No	4.1^c^	6.9^b^	6.8^b^	7.6^ab^	7.8^a^	8.0^a^	**
Price of kits, L.E	90	90	90	90	90	90	––
Net profit, L.E	369^e^	621^d^	612^d^	684^c^	702^b^	720^a^	**
Total feed intake, kg	14.72^a^	14.05^c^	14.25^b^	13.89^d^	14.22^b^	14.01^c^	**
Price of 1 kg feed, L.E	14.9	14.9	14.9	14.9	14.9	14.9	–-
Total feed cost/rabbit, L.E	219.3^a^	209.3^c^	212.3^b^	206.9^d^	211.9^b^	208.7^ cd^	**
Cost of treatment at weaning, L.E	0.00	6.00	6.00	9.00	2.4	23.4	–-
Total cost at weaning, LE	309.5^b^	305.3^b^	308.3^b^	305.9^b^	304.3^b^	322.1^a^	*
Net revenue, L.E	59.5^c^	315.7^b^	303.7^b^	387.1^a^	397.7^a^	397.9^a^	**
Economic efficiency, %	19.22^d^	103.41^c^	98.51^c^	126.54^b^	130.69^a^	123.54^b^	**

^a^^,^^b^Means in the same row with different letters are significantly different. SEM: Mean standard error NS: Non-significant; *: (*P* ≤ 0.05) and **: (*P* ≤ 0.01). Net profit = Litter size at weaning × price of kits;

Net revenue = Net profit – total cost at weaning; Economic efficiency = (Net revenue / Total cost at weaning*)* × 100; Price of Hb/kg = 75 LE; Price of DPP/kg = 400 LE; Price of BP/kg = 500 LE and Price of synbiotic/kg = 200 LE.; Calculations included period from insemination of the doe to weaning; all prices of kits and feeds were calculated according to the prices in 2023

## Discussion

Honey bees secrete honey, venom, wax, bee pollen, bee brood, bee bread, and royal jelly with various nutritive and nutraceutical properties. El-Hanoun et al. (2007) reported that bee pollen significantly increased rabbit milk yield. Moreover, recently El-Sabrout et al. (2023) indicated the importance of bee venom to enhance the intestinal health of rabbits and motivate the digestive enzymes to best utilize its diets. Further, it has been shown that bee products enhanced the reproductive parameters of male rabbits (Suleiman et al. 2021). Additionally, giving diabetic male rabbits with honey bees improved metabolic attributes of carbohydrates and lipids (Shikoo and Bakeel 2021). In the current study honey bees and honey pollens remarkably improved all tested traits. In honey bee, three phenolic compounds were characterized, these are; catechin, caffeic acid and vanillic acid; however, in bee pollens six phenolic compounds were identified, such as hesperidin, cinnamic acid, apigenin, rutin, chlorogenic acid, and kaempferol. These compounds owe antioxidant, digestive, antimicrobial, immunostimulatory, nutraceutical, and growth promoting properties (Bakour et al. 2022).

Daisa et al. (2013) found that rabbit diets supplemented with BP resulted in a higher conception rate, a lower number of services per conception, a larger litter size, and higher milk production. In addition, Attia et al. (2015) recorded fewer services per conception and a greater fertility rate by administering bee pollen as a growth stimulant than the control group. According to Ghanbari et al. (2018) the high micronutrient content of bee pollen is attributable to polyunsaturated fatty acids, minerals, vitamins, amino acids, and the bioactivities of flavonoids, carotenoids, and phenolic compounds. This composition enhanced rabbit doe reproductive performance, milk production, and offspring growth performance (Kolesarova et al. 2013). Likewise, Abdel-Hamid and El-Tarabany (2019) recorded that supplementation with BP increased BW and average daily gain. In a similar study, Zeedan et al. (2017) found that growing rabbits treated with BP improved final body weight and total weight gain while diminishing feed intake and improving feed conversion as compared with the control group. Similarly, Zeedan and El-Neney (2014) documented that supplementation with BP improved rabbit’s performance, BWG, reduced feed intake, and improved FCR. In a recent meta-analysis review, Sierra-Galicia et al. (2023) reported that rabbit diet enrichment with bee pollens decreased feed intake, and feed conversion ratio, serum creatinine, AST, and ALT; however, it increased average daily gain, and hot carcass yield. Provisions of the mammalian body with such rich-nutrients compounds are most likely capable for compensation of regular feedstuffs and meeting the body requirements.

Concerning probiotics, prebiotics and synbiotics, Elena Kurchaeva et al. (2020) stated that consumption of synbiotic had a positive effect on the productive index of rabbits; i.e., live weight, and average daily gain. In harmony with our results, Bansal et al. (2011) recorded that probiotic supplementation to broiler diets enhanced the digestive tract, leading to increased body weight and feed conversion ratio in a physiologically natural way, as well as improved digestion by balancing the resident gut bacteria. Prebiotics and probiotics, according to Ashayerizadeh et al. (2009), are growth promoters that can be administered as feed additives since they improve broiler’s performance. Similarly, Abdelhady and El-Abasy (2015) indicated that rabbits received mixture of prebiotic and probiotic (synbiotic) revealed the highest value of daily weight gain and the best feed conversion ratio (FCR). Lam and Jamikorn (2017) recorded that those rabbits fed probiotic (*Bacillus subtilis* and *Lactobacillus acidophilus*) had an increased body weight gain, greater growth and better feed conversion ratio than control. Similar results were obtained by El-Shafei et al. (2019) who revealed that rabbit diets containing a probiotic (*Lactobacillus planterium*) have positive effects (*P* ≤ 0.05) on both body weight and feed conversion ratio. Also, Chinwe et al. (2021) showed that rabbit diet supplementation with synbiotics significantly increased final body weight (FBW) and daily body weight gain compared to control diets. A similar study by Nesrein et al. (2021) recorded that growing rabbits fed with alginate-synbiotic led to significantly higher BWG and FCR than control. Heat stress imposes several physiological, nutritional, and behavioral disturbances on animals. Since rabbits are characterized by heavy fur coat, this impedes its ability to dissipate the excess body heat through sweat. In hot climates, raising rabbit necessitates several managerial facilities to mitigate the heat load, such as cooling sprinklers, air conditioning, and big fans. Even though, these strategies reduced the heat stress load on the animal, there is still a need for extra alternatives to maximize the rabbit productivity and minimize the mortality rate. Recently, Ebeid et al. (2023) in a comprehensive review documented that supplementation of probiotics, prebiotics, and synbiotic to rabbit diets raised in hot climates mitigates the negative impact of heat stress, improved antioxidant, immune system, digestion, growth, reproduction, and carcass quality. Abdelsalam et al. (2019) stated that the nutritional approach supported with synbiotic is a good way to alleviate the negative impact of heat stress on the rabbit’s productivity. Moreover, supplementing rabbit diets with probiotics inhibited the colonization and development of undesirable pathogens, but augmented the beneficial microorganisms that play indispensable roles in feed digestion, enzyme activity, volatile fatty acids production, vitamin synthesis, and antimicrobial peptide production (Mancini and Paci 2021). Parallel to the current findings, Ayyat et al. (1996) suggested that diets supplemented with live yeast (*Lacto-Sacc*) for lactating rabbits increased milk production, litter size, and weight at weaning. Furthermore, Belhassen et al. (2016) reported that the fertility rate and the viability rate of litter at birth of rabbit does were higher in the live yeast (probiotic) group than those with the control diet.

Regarding date palm pollen, several studies are currently investigating the use of herbal preparations for the treatment and prevention of infertility (Maham et al. 2021). The reason for using such a herbal compound is because it contains plethora of bioactive ingredients such as antioxidants, phenolics and flavonoids which were identified and separated from palm pollen (Abd El-Azim et al. 2015). Date palm pollens increased female fertility due to its contents of estrone- and estrogen-like compounds, sterols, and steroidal saponin glycosides (Moshfegh et al. 2016). Such steroid-like compounds may play vital roles in animal fertility. Maham et al. (2021) declared that date palm pollens improved reproductive performance in rabbits via enhancement of oogenesis, strengthening oocytes, hormone regulation, augmenting pregnancy, and the prevention of uterine rupture. Additionally, Taghian et al. (2017) noted that DPP exhibited higher total feed intake than the control resulting in higher body weight and average daily gain.

Alternatively, Baagar et al. (2022) examined the effect of oral administration of two levels of DPP to female rabbits and concluded that DPP elevated estrogen, progesterone, prolactin, ovulation rate, litter survival rate, pregnancy rate, litter weight at birth and weaning.

With respect to thyroid hormones (T3 and T4), there were significant increases in the levels of both hormones in broilers given bee products (i.e., bee pollens, propolis) during 7- 42 days of age (Rabie et al. 2018). Additionally, supplementing hypercholesterolemic rats with lactobacillus-based probiotics elevated serum T3 and T4 (Awaisheh et al. 2013). In diabetic rats, T3 and T4 levels were lower than in healthy animals, however supplementing these diabetic rat’s diets with bee and palm pollens alleviated the levels of T3 and T4 to approach the control ones (Mohamed 2018). El-Shafei et al. (2019) recorded that supplementing growing rabbits with probiotics increased concentrations of T_3_ and T_4_. Apparently, the positive effect of probiotics on T_3_ and T_4_ values in the present study could be explained by a higher absorptive capacity of the intestinal mucosa due to histo-morphological changes (Aliakbarpour et al. 2012) and/or a more effective digestion of the diet due to higher intestinal enzyme activity (Wang and Gu 2010), thus increasing the nutrients available to the animals.

Suh-Ching et al. (2005) recorded that rat’s intake of a synbiotic mixture significantly improved the ecosystem of the intestinal tract by increasing the probiotic population and the digestive enzymes such as lipase, lactase, sucrase, and iso-maltase activities. Also, Wang et al. (2008) found that probiotic intake can improve the condition of the digestive tract, which is short on digestive enzymes. Interestingly, Zeedan et al. (2017) showed that the treated rabbit's diet with BP significantly increased pancreatic homogenate and intestinal contents of activity of lipase, amylase and protease than that of the control. This improvement at high BP levels in the activity of enzymes may be due to the intake role in the overall digestive kinetic action causing the titers to rise of digestive enzymes in pancreatic tissue and intestinal content. Taghian et al. (2017) explained that the enhanced growth performance due to the addition of DPP could be associated with higher amino acid absorption or/and antibacterial activities of DPP enzymes or coenzymes. Also, in rabbits treated with BP, enhanced digestive enzyme activity and intestinal mucosa morphology may be partially responsible for the increased growth rate.

Nutrient digestibility and nutritive values were improved in the rabbits that received the tested compounds. Probiotics are a mixture of enzymes released from anaerobic bacteria that enhance the digestion of low-quality roughage (Gado et al. 2017). In harmony to the current findings, Abdel-Wareth et al. (2021) obtained better digestibility coefficients of the rabbit diet’s nutrients by providing probiotics blended with enzymes. Likewise, live yeast and its extracts were an effective solution in rabbit diets to counteract the harmful effect of heat stress, in addition it enhanced the nutrients digestibility coefficients (Abd El-Aziz et al. 2021). These improvements are most probably due to colonization of probiotics in the hind gut, contributing to establishment of balanced microflora and, as a response, protect the intestinal epithelium against pathogens (Bhatt et al. 2017). Thus, the higher digestibility in the treated groups in the present study could be the result of enhanced gastrointestinal health, bowel environment, and enzyme activities resulting in enhanced nitrogen usage and efficient FCR (Mateos et al. 2006). In conclusion, the ultimate goal of the study is to maximize the productivity and reproductive efficiency of female rabbits, which apparently was achieved by all selected bioactive ingredients. However, the mixture of the four selected compounds resulted in the best survived offspring with highest weaning weights and resulted in the best revenue. A comprehensive study is warranted to focus on the roles played by each active constituent in the tested compounds to monitor their exact mechanisms.

## Data Availability

The datasets generated during and/or analyzed during the current study are not publicly available due to the regulations of the employer institute, but are available from the corresponding author on reasonable request.

## Declarations

### Human and animal right

The study was approved by the animal rights and ethical use committee of the Animal Production Research Institute (APRI), Ministry of Agriculture and Land reclamation, Egypt in accordance with the Declaration of Helsinki.
